# A hydrogen-bonded channel structure formed by a complex of uracil and melamine

**DOI:** 10.1186/1860-5397-3-17

**Published:** 2007-05-23

**Authors:** Reji Thomas, G U Kulkarni

**Affiliations:** 1 Chemistry and Physics of Materials Unit, Jawaharlal Nehru Centre for Advanced Scientific Research, Jakkur (P.O), Bangalore – 560064, India

## Abstract

The structure of a 1:1 complex of uracil and melamine obtained by cocrystallization has been investigated. The structure involves hydrogen bonded layers with apertures stacked along the *a* and *b* directions giving rise to channels, unlike the complex of cyanuric acid with melamine.

## Background

Hydrogen bonded organic structures have emerged to become an important area of research because of the wide variety of fascinating structural features as well as properties exhibited by them. Of the many interesting hydrogen bonded systems, those formed by cyanuric acid and melamine are specially interesting. Thus, cyanuric acid forms hydrogen bonded adducts with melamine, [1] 4,4'-bipyridyl [2] and 4,4'-bipyridylethene. [3] The last two involve layered structures, but that with melamine is fascinating and involves a rosette type structure wherein each molecule of cyanuric acid and melamine forms three hydrogen bonds on either side. Such a structure of the 1:1 molecular complex between cyanuric acid and melamine first proposed by Whitesides, [4] was synthesized under hydrothermal conditions. [1] The rosette structure gives rise to channels in the perpendicular direction. Trithiocyanuric acid also forms a similar rosette structure with melamine. [1] Besides, trithiocyanuric acid forms a novel hydrogen-bonded channel structure with 4,4'-bipyridyl. [5] Although hydrogen bonding aspects of cyanuric acid have been studied in detail, similar structures formed by uracil possessing two imide groups has not been studied adequately, except for its hydrogen bonding with adenine in RNA. [6] In RNA, uracil forms cyclic dimers comprising of N-H...O and N-H...N bonds with adenine. The 1:1 molecular complex of 9-ethyladenine and N-methylcyanuric acid has a hydrogen-bonded layer structure. [7] In view of the above, it was our interest to study the hydrogen-bonded structure of cocrystals of uracil and melamine, with the expectation that the structure may involve channels as in the reported structure of the 1:1 molecular complex of cyanuric acid and melamine. In this article, we describe the structure of the hydrogen bonded adduct of uracil and melamine, and bring out the role of uracil in molecular recognition.

**Scheme 1:**
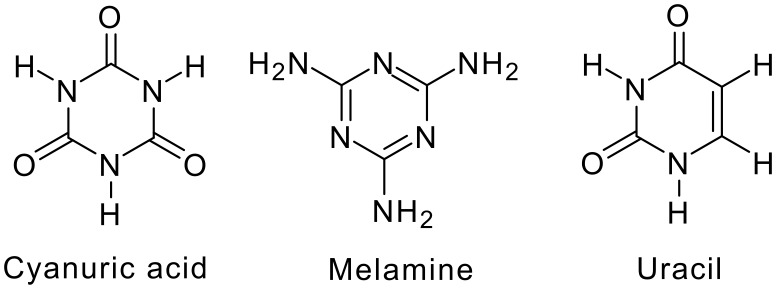
Molecules under discussion.

## 

Rod-shaped crystals of the 1:1 molecular complex of melamine and uracil were obtained by the slow evaporation of an equimolar solution of the two compounds in methanol at room temperature. The crystal data was collected on a Bruker-Nonius diffractometer with Kappa geometry attached with an APEX-II CCD detector. The intensity data were processed using SAINT [8] software of the Bruker suit programs. The structures were solved and refined using SHELXTL package [9]. The structure was solved by direct methods and refined by full-matrix least-squares techniques. All hydrogen atoms were located from a difference Fourier map (see Table 1 and Supporting Information File 1). The intermolecular interactions were analyzed using PLATON package [10] and are listed in Table 2.

**Table 1:** Crystal structure data.

Molecular complex	Melamine + Uracil
Empirical formula	C_3_H_6_N_6_·C_4_H_4_N_2_O_2_
Formula wt.	238.23
Temperature (K)	298 (2)
Crystal system	Monoclinic
Space group	C2/c
*a* (Å)	14.3618(10)
*b* (Å)	7.5472(6)
*c* (Å)	18.9922(14)
*β* (°)	91.838(4)
Volume (Å^3^)	2057.5(3)
*Z*	8
D_calc_ (Mg/m^3^)	1.538
*F*(000)	992
Crystal dimensions	0.27 × 0.22 × 0.15
*θ* range for data collection (°)	2.15 – 25.39
No. of measured reflections	4727
No. of unique reflections	1861
R_1_	0.0373
wR_2_	0.1053
Goof	1.043
CCDC No.	626355

**Table 2:** List of intermolecular interactions in melamine-uracil adduct

D-H...A	D-H	H...A/Å	D...A/Å	∠D-H...A/°	Symmetry

N1-H1...N7	0.90(2)	1.93(2)	2.833(2)	174.3(1)	1/2-x, 1/2+y, 1/2-z
N2-H2...N6	0.90(2)	1.98(2)	2.876(2)	178.7(2)	1/2-x, 3/2-y, -z
N4-H5...O2	0.84(2)	2.19(2)	3.004(2)	165.4(1)	1/2-x, -1/2+y, 1/2-z
N4-H6...N8	0.82(2)	2.27(2)	3.079(2)	169.2(2)	-x, y, 1/2-x
N3-H7...O1	0.91(2)	2.03(2)	2.941(2)	177.3(2)	1/2-x, 3/2-y, -z
N3-H8...N8	0.90(2)	2.50(2)	3.328(2)	153.6(1)	1/2-x, 1/2+y, 1/2-z
N5-H9...O2	0.86(2)	2.08(2)	2.937(2)	172.3(2)	1/2-x, 3/2-y, -z
N5-H10...O1	0.87(2)	2.02(2)	2.860(2)	164.2(2)	-x, 1-y, -z

**Figure 1:**
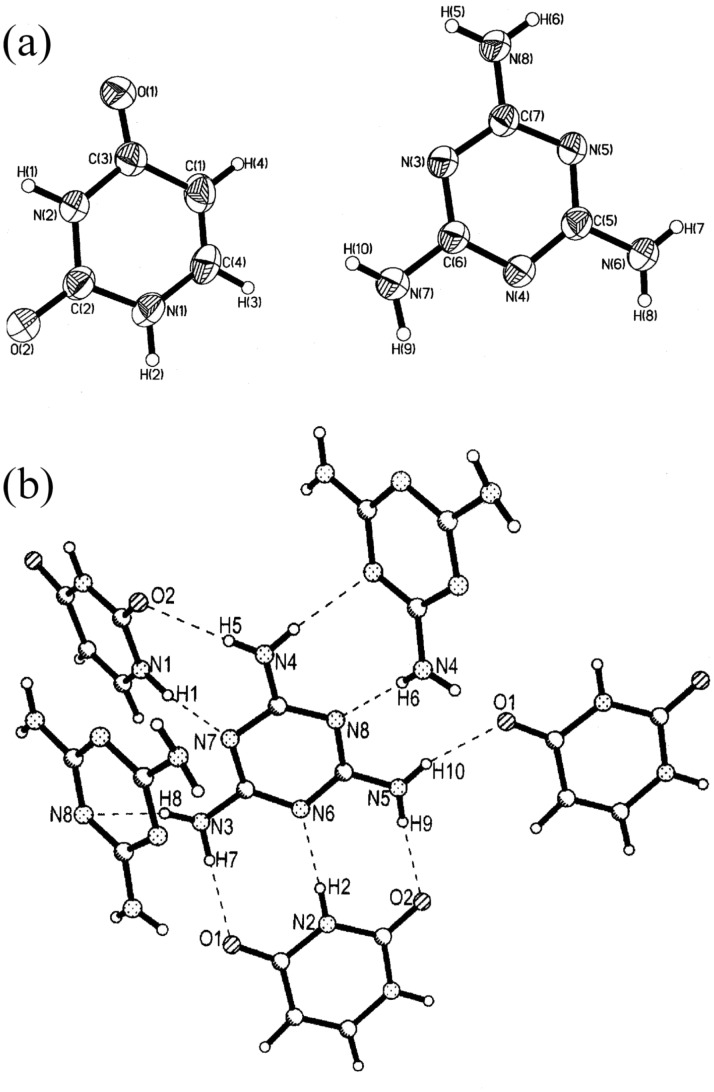
(a) ORTEP drawing of the asymmetric unit of the 1:1 molecular complex of uracil and melamine. (b) Unique hydrogen bonds present in the complex.

**Figure 2:**
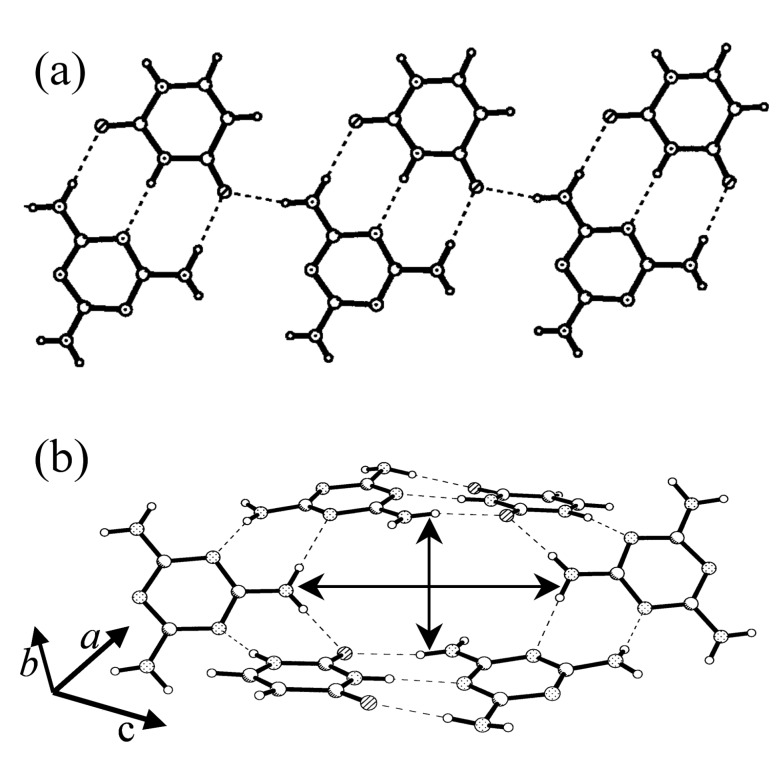
(a) Molecular chain formed by melamine and uracil molecules, (b) the aperture formed by the triply hydrogen-bonded melamine-uracil pairs.

**Figure 3:**
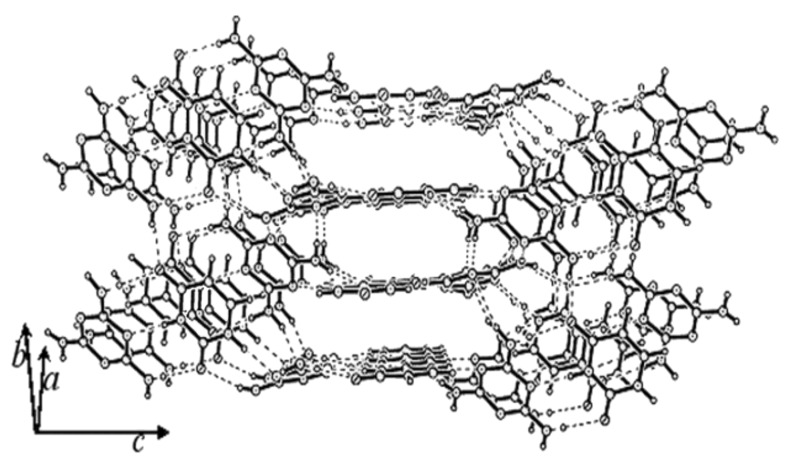
Three-dimensional packing of melamine and uracil molecules.

The crystal structure determination gave the asymmetric unit as shown in Figure 1a. The molecular complex crystallizes in C2/c space group with single molecules of melamine and uracil in the asymmetric unit. In this 1:1 complex, both melamine and uracil offer several donor-acceptor sites for hydrogen bonding and each melamine molecule engages in hydrogen bonding via N-H...O and N-H...N bonds with three uracil and two melamine molecules, as shown in Figure 1b (see Table 2). It is clear that the amino groups of the melamine molecule act as hydrogen bond donors and ring nitrogens as acceptors. Unlike with adenine, uracil forms two N-H...O bonds (H9....O2, 2.08Å; H7...O1, 2.03Å) on either side of the N-H...N bond (H2...N6, 1.98Å). All the three hydrogen bonds are strong, with the hydrogen bond angles in the range of 172 – 179°. These hydrogen-bonded pairs are interconnected through a N-H...O hydrogen bond to form a molecular chain as shown in the Figure 2a. The triply bonded melamine-uracil pairs are stacked parallely and the chains run along both *a* and *b* axes. Such an arrangement of the chains gives rise to an aperture structure as shown in Figure 2b. Four melamine and two uracil molecules thus enclose the aperture. The apertures are connected through N-H...O (H10...O1, 2.02Å) bonds giving rise to infinite channels along both *a* and *b* directions. We show one perspective in Figure 3, where the channels run along the *a*-direction. It is interesting to note that melamine engages in hydrogen bonding utilizing all the donor-acceptor sites, much the same way it does in the cyanuric acid adduct. [1] However, the channel structure is somewhat different, in shape and dimension. This clearly indicates the role of the uracil molecule in directing the shape of the channel structure.

In conclusion, we have illustrated the molecular recognition process of melamine with nucleobase uracil. We have also established the presence of channels, in the three-dimensional supramolecularly hydrogen-bonded structure of this adduct.

## Supporting Information

File 1 The data provided represent the structural and refinement details of the melamine-uracil crystal.
